# Duration in Immigration Detention and Health Harms

**DOI:** 10.1001/jamanetworkopen.2024.56164

**Published:** 2025-01-24

**Authors:** Altaf Saadi, Caitlin Patler, Paola Langer

**Affiliations:** 1 Department of Neurology, Harvard Medical School, Massachusetts General Hospital, Boston, Massachusetts; 2 Goldman School of Public Policy, University of California, Berkeley

## Abstract

**Importance:**

Length of custody is a mechanism by which carceral systems can worsen health. However, there are fewer studies examining US immigration detention, in large part because US immigration detention is largely privately operated and opaque by design.

**Objectives:**

To examine the association between duration spent in US immigration detention with subsequent health outcomes.

**Design, Setting, and Participants:**

This cross-sectional study used a referral sample of recently detained immigrants who were released from Immigrations and Customs Enforcement (ICE) under a series of court orders in 2020 and 2021. Data were analyzed from June 2023 to October 2024.

**Exposure:**

Detention duration less than 6 months vs 6 months or longer.

**Main Outcomes and Measures:**

We used multivariable regression analysis to assess the association of detention duration and several variables: (1) self-rated health, (2) mental illness (Kessler 6-item psychological distress scale), and (3) posttraumatic stress disorder (PTSD) (Primary Care-PTSD-5 Screen). We then calculated the likelihood of experiencing each outcome, controlling for covariates. As a robustness check, we conducted sensitivity analyses with detention duration as a continuous measure.

**Results:**

The study included 200 respondents (mean [SD] age, 40.3 [10.1] years; 175 male [87.5%]; 149 Hispanic or Latino ethnicity [74.5%]). Results revealed a high prevalence of poor self-rated health, mental illness, and PTSD for all respondents, but especially among those who had been detained for 6 months or longer, who had significantly higher likelihood of poor or fair self-rated health (49.1% [95% CI, 40.5%-57.6%] vs 30.4% [95% CI, 21.8%-39.1%]; *P* < .001), mental illness (37.0% [95% CI, 28.2%-45.8%] vs 20.7% [95% CI, 12.6%-28.7%]; *P* < .001), and PTSD (59.3% [95% CI, 50.3%-68.3%] vs 34.8% [95% CI, 25.3%-44.3%]; *P* < .001). Sensitivity analysis confirmed the general robustness of these findings, with longer detention duration significantly associated with mental illness (OR, 1.11 [95% CI, 1.02-1.20]; *P* = .01) and PTSD (OR, 1.11 [95% CI, 1.03-1.20]; *P* = .005) in our adjusted models.

**Conclusions and Relevance:**

In this cross-sectional study, detained immigrants experienced a high prevalence of poor health, mental illness, and PTSD, with detention periods of 6 months or more associated with higher rates compared with those detained less than 6 months. Duration of custody is one mechanism by which immigration detention might be a catalyst for worsening health.

## Key Points

**Question:**

What is the association of duration of immigration detention and subsequent health outcomes?

**Findings:**

In this cross-sectional study of 200 recently detained US immigrants, there was a high prevalence of poor self-rated health, mental illness, and posttraumatic stress disorder (PTSD) for all, but especially among those who had been detained for 6 months or longer, who had a significantly higher likelihood of poor or fair self-rated health, mental illness, and PTSD.

**Meaning:**

These results suggest that the duration of custody is one mechanism by which immigration detention can serve as a catalyst for worsening health.

## Teaser

This cross-sectional study of recently detained US immigrants examines the association of detention duration of 6 months or more with poor health, mental illness, and posttraumatic stress disorder.

## Introduction

Incarceration contributes to higher mortality and worse physical and mental health outcomes, including increased rates of communicable and chronic disease.^1^ Although few studies examine detention in the US immigration context, largely because US immigration detention is predominately privately run and opaque by design, similar health harms have also been demonstrated in this parallel system.^2,3^ Immigration detention in the US de facto mirrors the punitive conditions of prisons under criminal law, albeit de jure the system operates under civil law and therefore is legally defined as nonpunitive.

Numerous mechanisms can produce worsening health in carceral settings. Direct harms can include delayed access to care and subpar quality of care in a reactive rather than preventive medical care system,^2^ while indirect harms can include meals with insufficient nutritional value, sleep deprivation, isolation from family and support systems, and physical and psychological threats to safety including abuse by guards, and disciplinary practices such as solitary confinement.^3^ These conditions often co-occur and exert cumulative harm,^3^ often against a backdrop of preexisting trauma experienced by many people in immigration detention, pre- and peri-migration, that can heighten their vulnerability to the harms of incarceration.^4^

Immigration detention facilities and deportation proceedings operate under civil law; consequently, detained people lack some protections available under criminal law. For example, there are few limits on the length of detention and no systemic mechanism for release such as bail. In this context, prolonged, indefinite immigration detention is the norm for those fighting their removal cases. As of December 2023, the average length of detention in Immigration and Customs Enforcement (ICE) custody was about 52 days, although immigrants who are deported within a short time frame skew this data^5,6^; data from a class action lawsuit challenging mandatory detention found that immigrants who applied for relief from removal were held in California ICE detention facilities for an average of 421 days.^7^ A 2022 probability sample of 306 Mexican nationals deported from the US found that 47% had experienced detention lasting more than a year.^8^

Length of custody is one mechanism by which carceral systems can serve as a catalyst for worsening health. Both in the criminal and immigration contexts, increased length of imprisonment increases exposure to a subpar health system that directly exerts harm, and to conditions of confinement that directly and indirectly inflict harm. However, while studies from other countries with mandatory and indefinite immigration detention, such as Australia, find a link between longer detention and worse mental health outcomes, few US studies have examined this link.^9,10,11^ This study extends research into the impact of prolonged detention by assessing the association of duration in US immigration detention with subsequent health outcomes.

## Methods

### Study Design and Sample

We analyzed telephone survey data from immigrants who were detained by US Immigration and Customs Enforcement (ICE) and then released in the US under a series of court orders during the COVID-19 pandemic in 2020 and 2021. The American Civil Liberties Union (ACLU), which led these court cases, provided contact information for 355 individuals released under court order. At least 15 attempts were made to contact each individual, with the following results: 221 (62.3%) completed full surveys, 44 (12.4%) were ineligible, 21 (5.9%) declined to participate, and 52 (14.6%) could not be reached. We have no information about the characteristics of individuals who did not complete the survey so cannot assess how closely they resemble our study participants. Study materials and procedures were approved by the institutional review boards of the University of California, Davis and Massachusetts General Hospital. Participants provided verbal informed consent. Analysis of deidentified survey data was determined exempt by the institutional review board of the University of California, Berkeley.

Participants were residing in 27 states, with 157 (78.5%157) located in California. Because ICE does not release health information about the population it detains, this study cannot—and was not designed to—represent the entire detained population. Instead, the study provides an in-depth exploration of the health of a group of individuals who experienced immigration detention, one of few studies that can examine the health of recently detained immigrants.^8,12,13,14,15,16^

Survey materials were adapted from studies of formerly imprisoned people and detained immigrants^13,17,18,19^ and customized for a focus on health outcomes. The 93-question survey asked about health and access to care before, during, and after detention; demographic background; work and legal history; family and household composition; and detention and postrelease experiences. Surveys were conducted in English or Spanish after participants provided informed consent, lasted 48 minutes on average (median length, 45 minutes [range, 24-96 minutes]), and participants received a $10 gift card for participation as well as entry into a raffle for an Apple iPad. This study adhered to the Strengthening the Reporting of Observational Studies in Epidemiology (STROBE) reporting guideline for cross-sectional studies.

### Outcome Variables

We assessed self-rated health after release from immigration detention, mental illness, and posttraumatic stress disorder (PTSD). Self-rated health (SRH) is a validated predictor of mortality, morbidity, and use of health services.^20,21^ Mental illness and PTSD, which are highly prevalent among detained populations, irrespective of asylum or refugee status,^18^ are also associated with high mortality rates and a variety of chronic physical health problems.^22,23^

#### Fair or Poor SRH After Release

To assess SRH after release from detention, we relied on a measure from the Patient-Reported Outcomes Measurement Information System (PROMIS).^24^ Study participants described their health during detention on a Likert scale of excellent, very good, good, fair, or poor. We created a binary measure of poor health (poor or fair health = 1; good, very good, or excellent health = 0).

#### Mental Illness

The Kessler 6-item (K6) psychological distress scale measured symptoms of psychological distress.^25^ Items are summed to obtain a total score ranging from 0 to 24.^25^ A K6 score of 13 or greater is a strong indicator of the presence of a diagnosable mental illness with considerable disability^26^; as such, we created a dichotomized measure of mental illness (K6 score 13 or higher vs below 13).

#### Posttraumatic Stress Disorder

We measured PTSD using the Primary Care-PTSD-5 Screen.^27^ We dichotomized the measure, which ranges from 0 to 5, to equal 1 if the score was equal to 5 and zero if the score was 0 to 4. The threshold of Primary Care-PTSD-5 score above 4 has shown high levels of diagnostic accuracy and is well accepted among general primary care patients.^27^

### Key Independent Variable

We calculated the length of custody by subtracting the release date from the date of apprehension by ICE. We then created a binary variable for detention length (1 = 6 months or more; 0 = less than 6 months). We focused on detention lasting 6 months or longer for 2 reasons. First, policy-relevant litigation such as *Jennings v Rodriguez* (a class action lawsuit that sought to provide a bond hearing to immigrants held in detention beyond 6 months) argues that prolonged detention lasting longer than 6 months raises constitutional concerns, requires heightened protections, and is particularly punitive. Second, in other Global North countries, reentry outcomes such as employment are significantly impacted by time served lasting 6 months or longer. Mental illness may also worsen with time in detention.^10,28^ As a sensitivity analysis, we analyzed a continuous measure of months of detention, ranging from 0 to 12 months, with values of 12 or more recoded to 12 months given the long-tailed distribution of the data.

### Covariates

We accounted for age (in years), self-reported gender (male = 1, female = 0), educational attainment (1 = high school or more, 0 = less than high school), self-reported Hispanic or Latino ethnicity (1 = Hispanic, 0 = not Hispanic), and having a criminal record (1 = has been convicted or pled guilty or no contest to a crime in the US, 0 = has not). We accounted for potential health selectivity with 2 predetention measures: health status in the year prior to detention (fair or poor = 1; good, very good, or excellent = 0), and health insurance access in the year prior to detention (1 = had insurance, 0 = did not have insurance).

### Statistical Analysis

Missingness ranged from 0% to 3.2% (0 to 7 cases) on the variables in our models and did not vary systematically; after listwise deletion, our effective sample included 200 individuals (90.5% of the full sample of 221 respondents). For descriptive analyses, we used 2-tailed tests of means between respondents by detention length, using χ^2^ tests for binary variables and *t* tests for continuous variables. We then used logistic regression to model poor or fair SRH, mental illness, and PTSD. We used 2-sided hypothesis testing and an a priori significance level of *P* = .05. We also conducted Akaike Information Criterion (AIC) and Bayesian Information Criterion (BIC) tests of goodness of fit and parsimony of each model, wherein smaller AIC and BIC values represent better model fit. The likelihood ratio χ^2^ tests the difference in fit statistics between the fully adjusted model and the base model. A *P* value < .05 indicates that the fully adjusted model fits better than the base model for any given outcome variable. To further illustrate our results, we then calculated the estimated likelihood of experiencing each outcome variable based on the fully adjusted models, expressed as a percentage. We conducted all analyses using Stata version 16.0 (StataCorp). Data were analyzed from June 2023 to October 2024.

## Results

The sample comprised 200 participants (mean [SD] age, 40.3 (10.0) years) and was mostly male (75 participants [87.5%]) and Hispanic (149 [74.5%]), with most having a high school degree or higher (123 [61.5%]) and a criminal record (143 [71.5%]) (Table). The mean (SD) length of detention was 9.9 (11.2) months (median [IQR], 6.0 [3.0-12.0] months) and about half of participants were detained for 6 months or longer (108 participants [54.0%]). The mean time since release for the sample was 9.9 months (IQR, 8-12 months). Forty percent self-assessed their health as poor or fair prior to detention (80 participants [40.0%]), and approximately half had health insurance prior to detention (97 participants [48.8%]).

**Table.** Sample Characteristics by Detention Length

Characteristics	Respondents, No. (%)			*P* value^a^
	Total (n = 200)	Detention <6 mo (n = 92)	Detention ≥6 mo (n = 108)	
Age, mean (SD), y	40.3 (10.1)	39.4 (10.2)	41.1 (9.9)	.24
Sex				
Male	175 (87.5)	82 (89.1)	93 (86.1)	.20
Female	25 (12.5)	10 (10.9)	15 (13.9)	.20
Hispanic or Latino ethnicity	149 (74.5)	71 (77.2)	78 (72.2)	.48
High school degree or more	123 (61.5)	57 (62.0)	66 (61.1)	.66
Has a criminal record	143 (71.5)	66 (71.7)	77 (71.3)	.82
Detention length, mean (SD), mo	9. 9 (11.2)	2.9 (1.8)	15.8 (12.3)	<.001
<6 mo	92 (46.0)	92 (100.0)	0	<.001
≥6 mo	108 (54.0)	0	108 (100.0)	<.001
**Clinical characteristics**				
Predetention				
Poor or fair health	80 (40.0)	34 (37.0)	46 (42.6)	.56
Had health insurance	97 (48.5)	42 (45.7)	55 (50.9)	.37
Postdetention				
Poor or fair health	81 (40.5)	28 (30.4)	53 (49.1)	.01
Good, very good, or excellent health	119 (59.5)	64 (69.6)	55 (50.9)	.01
K6 as continuous, mean (SD)	9.5 (6.3)	8.4 (6.3)	10.4 (6.3)	.03
Mental illness (K6 score ≥13)	59 (29.5)	19 (20.7)	40 (37.0)	.01
PTSD (PC-PTSD ≤4)	96 (48.0)	32 (34.8)	64 (59.3)	<.001

Abbreviations: K6, Kessler 6-item; PC-PTSD, Primary Care-PTSD-5; PTSD, posttraumatic stress disorder.

^a^ Based on 2-tailed tests (χ^2^ for binary variables; *t* tests for continuous variables).

Nearly half of participants self-assessed their health as poor or fair after being released from detention (81 respondents [40.5%]). The mean (SD) K6 score was 9.5 (6.3) and approximately a third of participants experienced mental illness (59 [29.5%]), defined as having a K6 score of 13 or higher. Almost half of participants met the screening threshold for PTSD (96 participants [48.0%]).

In multivariable regression analyses, our analyses revealed very high rates of poor self-rated health, mental illness, and PTSD for all study participants, with a further, statistically significant health penalty for those detained 6 months or longer (Figure; eTable 1 in Supplement 1). Those who were detained for 6 months or longer had significantly higher likelihood of poor or fair SRH (49.1% [95% CI, 40.5%-57.6%] vs 30.4% [95% CI, 21.8%-39.1%] for those detained less than 6 months; *P* < .001), mental illness (37.0% [95% CI, 28.2%-45.8%] vs 20.7% [95% CI, 12.6%-28.7%]; *P* < .001), and PTSD (59.3% [95% CI, 50.3%-68.3%] vs 34.8% [95% CI, 25.3%-44.3%]; *P* < .001). Our sensitivity analysis of the continuous detention length measure (0-12 months or more) showed a similar pattern, with longer detention duration significantly associated with mental illness (OR, 1.11 [95% CI, 1.02-1.20]; *P* = .01) and PTSD (OR, 1.11 [95% CI, 1.03-1.20]; *P* = .005) in our adjusted models (eTable 2 in Supplement 1). There was also an association with SRH in the unadjusted analysis (OR, 1.08 [95% CI, 1.00-1.16]; *P* = .04) but not in the adjusted analysis (OR, 1.08, [95% CI, 1.00-1.17]; *P* = .05).

**Figure.**
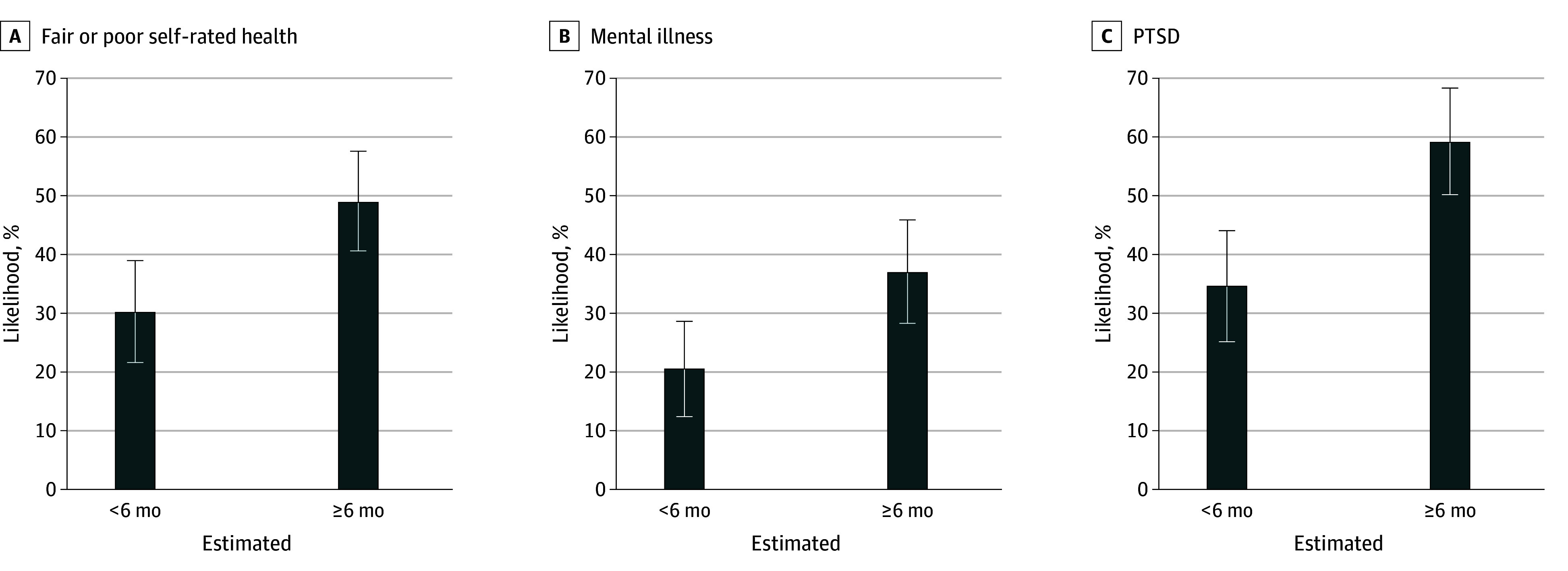
Estimated Health Outcomes by Detention Length Estimated values based on fully adjusted models presented in eTable 1 in Supplement 1. Mental illness is defined as a Kessler 6-item score of 13 or higher. Error bars indicate 95% CIs.

## Discussion

Our study provides evidence that poor self-reported health, mental illness, and PTSD were highly prevalent among all recently detained individuals, but that detention lasting 6 months or longer was associated with even higher rates of poor SRH, mental illness, and PTSD following release. These findings support existing empirical evidence that longer detention may worsen health and psychological distress.^9,10,11^ In sensitivity analyses, we also find significant associations between a continuous measure of time in detention and unadjusted and adjusted models predicting mental illness and PTSD, and in unadjusted models predicting self-rated health (SRH). Our study compliments results from a 2003 US-based survey of 70 asylum seekers detained in New York, New Jersey, and Pennsylvania in 2001 and 2002, which found an association between length of detention and clinically significant symptoms of anxiety, depression, and PTSD.^16^ Given the lack of recent research from the US, our study thus provides a novel and important contribution to understanding the association between US immigration detention and health.

Our findings provide evidence of a dose-response relationship between length of immigration detention and health harms. This is consistent with several studies from outside the US.^11^ In a 2006 study of 241 Iraqis and Syrians in Australia, those who spent more than 6 months in detention scored higher on measures of depression, PTSD, and mental health–related disability, although both groups with short- and long-term detention reported substantial stress.^10^ Another study among Mexican migrants deported from the US documented residual mental health harms from US immigration detention following release, most pronounced among those detained for longer.^8^

Our results are also consistent with studies in the US criminal context. In one study involving a national US sample, length of incarceration increased the likelihood of mental health symptoms and depression for formerly incarcerated persons, but not among those currently incarcerated.^29^ Another study documented an association between length of incarceration and mortality among imprisoned populations.^30^ Persistent mental health symptoms following release may also be influenced by psychosocial challenges faced upon reentry into society including but not limited to stigma, discrimination, and financial strain from difficulty securing employment following release.^28,31,32^

We found a high prevalence of poor self-rated health, mental illness, and PTSD among our entire sample, even among those detained for shorter periods. This is consistent with a 2013 Canadian study, which found that even detention lasting less than 1 month was associated with negative mental health consequences.^33^ The high prevalence is particularly stark when compared with US general population estimates. For example, whereas fewer than 17% of US adults judge their health to be poor or fair,^34^ 40.5% of our total sample did after release from detention. Whereas approximately 3% to 4% of US adults have mental illness (K6 scores of 13 or higher),^35^ 29.5% of our total sample did. Furthermore, while the mean psychological distress score (K6 score) in our sample was 9.5, it is around 2.5 in the US general population.^35^ Therefore, although our findings reveal an association between length of detention and poorer health outcomes, the comparison with the noninstitutionalized US general population highlights the vulnerabilities of all detained immigrants, and especially those detained for longer periods of time.

These results have implications for legal practitioners and policymakers concerned with protecting the health of detained immigrants. First, our study suggests that prolonged detention is associated with increased risks to health. These findings suggest that efforts to establish systematic mechanisms for release (eg, bond hearings) could mitigate health harms. Examples of such efforts include *Jennings v Rodriguez* and similar litigation. Second, our findings support better implementation and expansion of the currently limited circumstances in which bond is available for highly vulnerable individuals after 6 months in detention, ie, those with serious mental illness rendered unable to represent themselves in court through the National Qualified Representative Program (NQRP), made possible due to the *Franco-Gonzalez v Holder* class action lawsuit and settlement. Our study suggests that detained immigrants making use of NQRP and *Franco* class members could face increased health harms if they are denied their 6-month bond hearing or denied bond and not released at this hearing. Third, our results also support an expansion of NQRP and the *Franco* ruling, as the health harms we document are not exclusive to people with serious mental disorders in immigration detention. Indeed, our study findings could inform an immigration judge’s decision to grant bond for release of any detained individual, by highlighting the detrimental impact on their health. Finally, that indefinite detention without a right to a bond hearing is legally sanctioned for the majority of detained immigrants—despite its health harms—underscores existing US immigration detention policy as an embodiment of structural racism that perpetuates health inequities among immigrants.^36^

### Limitations

This study has several limitations. First, a population-wide probabilistic sampling method was not feasible as neither ICE nor the private prison companies that dominate immigration detention operations release health information about the populations they detain. Furthermore, because US immigration law offers no systematic mechanisms for release from detention that could facilitate greater access to currently or recently detained immigrants, convenience or referral samples remain one of the few ways to directly assess the health of this population, but such samples may not represent the entire detained population. For example, although our sample included individuals across the US, the vast majority were from California. Prior studies suggest regional differences, including longer length of detention and worse immigration detention conditions in the US South.^15^ Our study may therefore underestimate health harms experienced by some detained individuals. Researchers should continue to advocate for greater data transparency, alongside adequate data privacy and protections.

Second, release of the individuals in our sample during the COVID-19 pandemic may have included people with higher rates of chronic conditions, including mental illness. However, we adjusted for this possibility in our models by including predetention health status. Still, this may introduce other confounders that we cannot account for (ie, people with mental illness might have lower self-regulatory skills). Third, there are limitations to self-reported data, which may be impacted by inadequate health literacy, desirability effect, observational bias, or recall bias, although we note that most prominent national health studies (eg, the National Health Interview Survey [NHIS] and the Behavioral Risk Factor Surveillance System [BRFSS]) collect self-reported health data, as well as studies of imprisoned populations (eg, the Survey of Prison Inmates). Third, our sample disproportionately included Latino and Hispanic men, and small sample sizes prevent further detection of differences by racial and ethnic identity. Existing research suggests differences in immigration prison conditions of confinement experiences based on racial and ethnic identity, such as increased exposure to solitary confinement among African and Caribbean immigrants.^37^ Studies in the criminal context have also shown differences in the relationship between time served and mental health symptoms by gender and racialized group.^38^ Future studies should assess how immigration detention harms may differ across groups, including by racialized groups, sexual orientation, indigeneity, and/or gender identity.

## Conclusions

In this cross-sectional study of recently detained US immigrants, detained immigrants experienced a high prevalence of poor health, mental illness, and PTSD after being released, with those who had been imprisoned for 6 months or longer experiencing higher rates of these outcomes compared with those detained less than 6 months. Duration of detention may therefore represent one mechanism for worsening health among detained immigrants. Our findings are relevant to recent and ongoing federal litigation efforts to determine whether people held in US immigration facilities, including legal permanent residents and people applying for asylum, should access bond hearings after 6 months. Our findings suggest that the current practice of detention is associated with health harms of all detained people, and especially those who are held for longer periods of time. Policymakers should urgently consider alternatives to detention to optimize health for detained immigrants and their communities.

## Notes

Supplement 1. **eTable 1.** Logistic Regression Predicting Health Outcomes Using Binary Length of Detention Variable at 6 Months **eTable 2.** Logistic Regression Predicting Health Outcomes Using Length of Detention Continuous Variable

Supplement 2.
Data Sharing Statement

## References

[1] Wildeman C, Wang EA. Mass incarceration, public health, and widening inequality in the USA. Lancet. 2017;389(10077):1464-1474. doi:10.1016/S0140-6736(17)30259-3 28402828

[2] Venters HD, Keller AS. The immigration detention health plan: an acute care model for a chronic care population. J Health Care Poor Underserved. 2009;20(4):951-957. doi:10.1353/hpu.0.0213 20168008

[3] Saadi A, Patler C, De Trinidad Young ME. Cumulative risk of immigration prison conditions on health outcomes among detained immigrants in California. J Racial Ethn Health Disparities. 2022;9(6):2518-2532. doi:10.1007/s40615-021-01187-1 34845673 PMC8628823

[4] Saadi A, De Trinidad Young ME, Patler C, Estrada JL, Venters H. Understanding US immigration detention: reaffirming rights and addressing social-structural determinants of health. Health Hum Rights. 2020;22(1):187-197. 32669800 PMC7348446

[5] Transactional Records Access Clearinghouse, Syracuse University. Length of time immigrants remain in detention grows in recent weeks. TRAC What’s New. Published December 2023. Accessed April 4, 2024. https://trac.syr.edu/whatsnew/email.231222.html

[6] American Immigration Council. Immigration detention in the United States by agency. American Immigration Council. Published January 2, 2020. Accessed November 15, 2023. https://www.americanimmigrationcouncil.org/research/immigration-detention-united-states-agency

[7] American Civil Liberties Union. Rodriguez, et al v. Robbins, et al—prolonged detention fact sheet. Published online May 9, 2014. Accessed November 15, 2023. https://www.aclu.org/documents/rodriguez-et-al-v-robbins-et-al-prolonged-detention-fact-sheet

[8] Bakely L, Correa-Salazar C, Rangel Gómez MG, . Exploring the association between detention conditions, detention-related abuse, and mental health among deported Mexican migrants. J Health Care Poor Underserved. 2023;34(3):1021-1036. doi:10.1353/hpu.2023.a903060 38009112 PMC10671122

[9] Essex R, Kalocsányiová E, Young P, McCrone P. Psychological distress in Australian onshore and offshore immigration detention centres from 2014-2018. J Immigr Minor Health. 2022;24(4):868-874. doi:10.1007/s10903-022-01335-7 35113325 PMC9256570

[10] Steel Z, Silove D, Brooks R, Momartin S, Alzuhairi B, Susljik I. Impact of immigration detention and temporary protection on the mental health of refugees. Br J Psychiatry. 2006;188(1):58-64. doi:10.1192/bjp.bp.104.007864 16388071

[11] von Werthern M, Robjant K, Chui Z, . The impact of immigration detention on mental health: a systematic review. BMC Psychiatry. 2018;18(1):382. doi:10.1186/s12888-018-1945-y 30522460 PMC6282296

[12] Diaz C, Ortiz V, Sanchez L, . Harmful by design-a qualitative study of the health impacts of immigration detention. J Gen Intern Med. 2023;38(9):2030-2037. doi:10.1007/s11606-022-07914-6 36451013 PMC9713141

[13] Patler C, Saadi A, Young MEDT, Franco K. Release from US immigration detention may improve physical and psychological stress and health: results from a two-wave panel study in California. SSM Ment Health. 2021;1:100035. doi:10.1016/j.ssmmh.2021.100035

[14] Patler C, Saadi A, Arulanantham A. Looking back: decarcerating immigration prisons as a tool for improved health. Am J Public Health. 2023;113(7):732-735. doi:10.2105/AJPH.2023.307293 37053530 PMC10262252

[15] Zeidan AJ, Goodall H, Sieben A, Parmar P, Burner E. Medical mismanagement in Southern US immigration and customs enforcement detention facilities: a thematic analysis of secondary medical records. J Immigr Minor Health. 2023;25(5):1085-1097. doi:10.1007/s10903-023-01451-y 36715966 PMC9885057

[16] Keller AS, Rosenfeld B, Trinh-Shevrin C, . Mental health of detained asylum seekers. Lancet. 2003;362(9397):1721-1723. doi:10.1016/S0140-6736(03)14846-5 14643122

[17] Patler C, Branic N. Patterns of family visitation during immigration detention. RSF Russell Sage Found J Soc Sci. 2017;3(4):18. doi:10.7758/rsf.2017.3.4.02

[18] Patler C, Saadi A. Risk of poor outcomes with COVID-19 among U.S. detained immigrants: a cross-sectional study. J Immigr Minor Health. 2021;23(4):863-866. doi:10.1007/s10903-021-01173-z 33661415 PMC7930890

[19] Western B, Braga A, Kohl R. A longitudinal survey of newly-released prisoners: methods and design of the Boston reentry study. Fed Probat. 2017;81:32.

[20] Idler EL, Benyamini Y. Self-rated health and mortality: a review of twenty-seven community studies. J Health Soc Behav. 1997;38(1):21-37. doi:10.2307/2955359 9097506

[21] DeSalvo KB, Fan VS, McDonell MB, Fihn SD. Predicting mortality and healthcare utilization with a single question. Health Serv Res. 2005;40(4):1234-1246. doi:10.1111/j.1475-6773.2005.00404.x 16033502 PMC1361190

[22] Thornicroft G. Premature death among people with mental illness. BMJ. 2013;346:f2969. doi:10.1136/bmj.f2969 23674141

[23] Felker B, Yazel JJ, Short D. Mortality and medical comorbidity among psychiatric patients: a review. Psychiatr Serv. 1996;47(12):1356-1363. doi:10.1176/ps.47.12.1356 9117475

[24] Hahn EA, DeWalt DA, Bode RK, ; PROMIS Cooperative Group. New English and Spanish social health measures will facilitate evaluating health determinants. Health Psychol. 2014;33(5):490-499. doi:10.1037/hea0000055 24447188 PMC4159098

[25] Kessler RC, Green JG, Gruber MJ, . Screening for serious mental illness in the general population with the K6 screening scale: results from the WHO World Mental Health (WMH) survey initiative. Int J Methods Psychiatr Res. 2010;19(Suppl 1)(suppl 1):4-22. doi:10.1002/mpr.310 20527002 PMC3659799

[26] Kessler RC, Barker PR, Colpe LJ, . Screening for serious mental illness in the general population. Arch Gen Psychiatry. 2003;60(2):184-189. doi:10.1001/archpsyc.60.2.184 12578436

[27] Prins A, Bovin MJ, Smolenski DJ, . The Primary Care PTSD Screen for DSM-5 (PC-PTSD-5): development and evaluation within a veteran primary care sample. J Gen Intern Med. 2016;31(10):1206-1211. doi:10.1007/s11606-016-3703-5 27170304 PMC5023594

[28] Ramakers A, Apel R, Nieuwbeerta P, Dirkzwager A, Van Wilsem J. Imprisonment length and post-prison employment prospects. Criminology. 2014;52(3):399-427. doi:10.1111/1745-9125.12042

[29] Porter LC, DeMarco LM. Beyond the dichotomy: Incarceration dosage and mental health. Criminology. 2019;57(1):136-156. doi:10.1111/1745-9125.12199

[30] Patterson EJ. Incarcerating death: mortality in U.S. state correctional facilities, 1985-1998. Demography. 2010;47(3):587-607. doi:10.1353/dem.0.0123 20879679 PMC3000056

[31] Frank JW, Wang EA, Nunez-Smith M, Lee H, Comfort M. Discrimination based on criminal record and healthcare utilization among men recently released from prison: a descriptive study. Health Justice. 2014;2:6. doi:10.1186/2194-7899-2-6 25642407 PMC4308970

[32] Schnittker J, John A. Enduring stigma: the long-term effects of incarceration on health. J Health Soc Behav. 2007;48(2):115-130. doi:10.1177/002214650704800202 17583269

[33] Cleveland J, Rousseau C. Psychiatric symptoms associated with brief detention of adult asylum seekers in Canada. Can J Psychiatry. 2013;58(7):409-416. doi:10.1177/070674371305800706 23870723

[34] Zajacova A, Dowd JB. Reliability of self-rated health in US adults. Am J Epidemiol. 2011;174(8):977-983. doi:10.1093/aje/kwr204 21890836 PMC3218632

[35] Tomitaka S, Kawasaki Y, Ide K, Akutagawa M, Ono Y, Furukawa TA. Distribution of psychological distress is stable in recent decades and follows an exponential pattern in the US population. Sci Rep. 2019;9(1):11982. doi:10.1038/s41598-019-47322-1 31427587 PMC6700099

[36] Diaz C, Nwadiuko J, Saadi A, Patler C. Advancing research to address the health impacts of structural racism in US immigration prisons. Health Aff (Millwood). 2023;42(10):1448-1455. doi:10.1377/hlthaff.2023.00479 37782876 PMC10966767

[37] Franco K, Patler C, Reiter K. Punishing status and the punishment status quo: solitary confinement in U.S. Immigration prisons, 2013–2017. Punishm Soc. 2022;24(2):170-195. doi:10.1177/1462474520967804

[38] Porter LC, Kozlowski-Serra M, Lee H. Proliferation or adaptation? Differences across race and sex in the relationship between time served in prison and mental health symptoms. Soc Sci Med. 2021;276:113815. doi:10.1016/j.socscimed.2021.113815 33812157

