# Supplementary material for: Duration in Immigration Detention and Health Harms
Source: JAMA Netw Open. 2025 Jan 24;8(1):e2456164. doi: 10.1001/jamanetworkopen.2024.56164 (PMC11762235; doi:10.1001/jamanetworkopen.2024.56164)
Supplement: Supplement 1. — eTable 1. Logistic Regression Predicting Health Outcomes Using Binary Length of Detention Variable at 6 Months eTable 2. Logistic Regression Predicting Health Outcomes Using Length of Detention Continuous Variable [file jamanetwopen-e2456164-s001.pdf]

Supplemental Online Content

Saadi A, Patler C, Langer P. Duration in immigration detention and health harms. *JAMA Netw Open.* 2025;8(1):e2456164. doi:10.1001/jamanetworkopen.2024.56164

eTable 1. Logistic Regression Predicting Health Outcomes Using Binary Length of Detention Variable at 6 Months

eTable 2. Logistic Regression Predicting Health Outcomes Using Length of Detention Continuous Variable

This supplemental material has been provided by the authors to give readers additional information about their work.

© 2025 Saadi A et al. *JAMA Network Open*.

eTable 1. Logistic Regression Predicting Health Outcomes Using Binary Length of Detention Variable at 6 Months

	Had poor/fair health post-detention (Ref.: good/very good/excellent)	Had poor/fair health post-detention (Ref.: good/very good/excellent)	Mental illness (K6 ≥13)	Mental illness (K6 ≥13)	PTSD (PC-PTSD>4)	PTSD (PC-PTSD>4)
Detention Length (Ref. <6 months)						
≥6 months	2.20**	2.36**	2.26*	2.27*	2.73***	2.80***
	(0.65)	(0.78)	(0.74)	(0.77)	(0.80)	(0.85)
	[1.23,3.94]	[1.23,4.52]	[1.19,4.28]	[1.17,4.41]	[1.53,4.85]	[1.54,5.09]
	[0.01]	[0.01]	[0.01]	[0.02]	[0.00]	[0.00]
Age in years		1.02		0.98		0.98
		(0.02)		(0.02)		(0.02)
		[0.98,1.05]		[0.95,1.01]		[0.95,1.01]
		[0.32]		[0.26]		[0.30]
Male (Ref.: Female)		0.51		1.49		0.51
		(0.25)		(0.77)		(0.25)
		[0.19,1.35]		[0.54,4.12]		[0.20,1.33]
		[0.17]		[0.44]		[0.17]
High School Degree or more (Ref.: Less than HS)		1.04		0.67		1.75
		(0.37)		(0.25)		(0.59)
		[0.52,2.07]		[0.32,1.38]		[0.90,3.39]
		[0.92]		[0.27]		[0.10]
Hispanic/Latina/o (Ref.: Not Latino)		1.96		0.59		1.03
		(0.81)		(0.23)		(0.37)
		[0.88,4.39]		[0.28,1.25]		[0.50,2.10]
		[0.10]		[0.17]		[0.94]
Has a criminal record (Ref.: No criminal record)		0.55		0.70		1.35
		(0.21)		(0.27)		(0.49)
		[0.26,1.17]		[0.34,1.47]		[0.67,2.75]
		[0.12]		[0.35]		[0.40]
		4.64***		1.59		1.78
		(1.55)		(0.55)		(0.57)
		[2.40,8.94]		[0.81,3.12]		[0.95,3.33]
		[0.00]		[0.18]		[0.07]
Had insurance pre-det		0.79		2.22*		1.26
		(0.26)		(0.76)		(0.39)
		[0.41,1.51]		[1.13,4.33]		[0.69,2.31]
		[0.47]		[0.02]		[0.45]
Constant	0.44***	0.21	0.26***	0.52	0.53**	0.71
	(0.10)	(0.18)	(0.07)	(0.47)	(0.12)	(0.60)
	[0.28,0.68]	[0.04,1.15]	[0.16,0.43]	[0.09,3.07]	[0.35,0.82]	[0.14,3.68]
	[0.00]	[0.07]	[0.00]	[0.47]	[0.00]	[0.69]
Observations	200	200	200	200	200	200
AIC	266.75	245.46	240.09	241.80	268.88	272.10
BIC	273.35	275.14	246.68	271.48	275.47	301.78
LR chi2		35.29***		12.29		10.77
		[0.00]		[0.09]		[0.15]

Notes: Table reports odds ratios from multivariable logistic regression with standard errors in parentheses, and the 95% Confidence Interval and p-values in brackets;

© 2025 Saadi A et al. *JAMA Network Open*.

* $p < 0.05$, ** $p < 0.01$, *** $p < 0.001$. Akaike Information Criterion (AIC) and Bayesian Information Criterion (BIC) provide tests of goodness of fit and parsimony of each model, wherein smaller AIC & BIC values represent better model fit. The Likelihood Ratio (LR) chi2 tests the difference in fit statistics between the fully adjusted model and the base model. A p-value≤0.05 indicates that the fully adjusted model fits better than the base model for any given outcome variable.

© 2025 Saadi A et al. *JAMA Network Open*.

eTable 2. Logistic Regression Predicting Health Outcomes Using Length of Detention Continuous Variable

	Had poor/fair health post-detention (Ref.: good/very good/excellent)	Had poor/fair health post-detention (Ref.: good/very good/excellent)	Mental illness (K6 ≥13)	Mental illness (K6 ≥13)	PTSD (PC-PTSD>4)	PTSD (PC-PTSD>4)
Detention length continuous capped at 12 months	1.08*	1.08	1.10*	1.11*	1.10**	1.11**
	(0.04)	(0.04)	(0.04)	(0.05)	(0.04)	(0.04)
	[1.00,1.16]	[1.00,1.17]	[1.02,1.19]	[1.02,1.20]	[1.03,1.18]	[1.03,1.20]
	[0.04]	[0.05]	[0.01]	[0.01]	[0.01]	[0.005]
Age (years)		1.02		0.98		0.99
		(0.02)		(0.02)		(0.02)
		[0.99,1.05]		[0.95,1.01]		[0.96,1.02]
		[0.29]		[0.26]		[0.34]
Male (Ref.: Female)		0.52		1.49		0.52
		(0.26)		(0.77)		(0.25)
		[0.20,1.36]		[0.54,4.09]		[0.21,1.34]
		[0.18]		[0.44]		[0.18]
High School Degree or more (Ref.: Less than HS)		1.04		0.67		1.74
		(0.36)		(0.25)		(0.58)
		[0.52,2.06]		[0.32,1.39]		[0.90,3.35]
		[0.91]		[0.28]		[0.10]
Hispanic/Latina/o (Ref.: Not Latino)		1.85		0.56		0.96
		(0.76)		(0.22)		(0.34)
		[0.83,4.12]		[0.26,1.20]		[0.47,1.94]
		[0.13]		[0.14]		[0.90]
Has a criminal record=1		0.59		0.77		1.48
		(0.22)		(0.29)		(0.53)
		[0.29,1.24]		[0.37,1.62]		[0.73,2.99]
		[0.16]		[0.49]		[0.28]
yes		4.72***		1.67		1.87*
		(1.57)		(0.57)		(0.59)
		[2.46,9.07]		[0.85,3.27]		[1.00,3.48]
		[0.00]		[0.14]		[0.05]
Had insurance pre-det		0.81		2.25*		1.30
		(0.27)		(0.77)		(0.39)
		[0.42,1.54]		[1.15,4.39]		[0.72,2.35]
		[0.52]		[0.02]		[0.39]
Constant	0.41**	0.18	0.21***	0.38	0.48**	0.54
	(0.12)	(0.16)	(0.07)	(0.35)	(0.14)	(0.46)
	[0.23,0.72]	[0.03,1.07]	[0.11,0.40]	[0.06,2.36]	[0.28,0.83]	[0.10,2.90]
	[0.00]	[0.06]	[0.00]	[0.30]	[0.01]	[0.47]
Observations	200	200	200	200	200	200

Notes: Table reports odds ratios from multivariable logistic regression with standard errors in parentheses, and the 95% Confidence Interval and p-values in brackets;
* $p < 0.05$, ** $p < 0.01$, *** $p < 0.001$. * $p < 0.05$, ** $p < 0.01$, *** $p < 0.001$

© 2025 Saadi A et al. *JAMA Network Open*.
