# Supplementary material for: Duration in Immigration Detention and Health Harms
Source: JAMA Netw Open. 2025 Jan 24;8(1):e2456164. doi: 10.1001/jamanetworkopen.2024.56164 (PMC11762235; doi:10.1001/jamanetworkopen.2024.56164)
Supplement: Supplement 2. — Data Sharing Statement [file jamanetwopen-e2456164-s002.pdf]

Data Sharing Statement

Data
Data available: No

Additional Information
Explanation for why data not available: Due to the sensitivity and legal implications of sharing the data, authors cannot widely release study data. Author CP is able to field requests for use of this data on an individual basis.
